# The development of socio-economic health differences in childhood: results of the Dutch longitudinal PIAMA birth cohort

**DOI:** 10.1186/1471-2458-11-225

**Published:** 2011-04-12

**Authors:** Annemarie Ruijsbroek, Alet H Wijga, Marjan Kerkhof, Gerard H Koppelman, Henriette A Smit, Mariël Droomers

**Affiliations:** 1 Centre for Prevention and Health Services Research, National Institute for Public Health and the Environment, Bilthoven, the Netherlands; 2 Department of Epidemiology and Bioinformatics, University of Groningen, Groningen, the Netherlands; 3 Department of Pediatric Pulmonology and Pediatric Allergology, University Medical Center Groningen, Groningen, the Netherlands; 4 Julius Center for Health Sciences and Primary Care, University Medical Center Utrecht, Utrecht, the Netherlands

## Abstract

**Background:**

People with higher socio-economic status (SES) are generally in better health. Less is known about when these socio-economic health differences set in during childhood and how they develop over time. The goal of this study was to prospectively study the development of socio-economic health differences in the Netherlands, and to investigate possible explanations for socio-economic variation in childhood health.

**Methods:**

Data from the Dutch Prevention and Incidence of Asthma and Mite Allergy (PIAMA) birth cohort study were used for the analyses. The PIAMA study followed 3,963 Dutch children during their first eight years of life. Common childhood health problems (i.e. eczema, asthma symptoms, general health, frequent respiratory infections, overweight, and obesity) were assessed annually using questionnaires. Maternal educational level was used to indicate SES. Possible explanatory lifestyle determinants (breastfeeding, smoking during pregnancy, smoking during the first three months, and day-care centre attendance) and biological determinants (maternal age at birth, birthweight, and older siblings) were analysed using generalized estimating equations.

**Results:**

This study shows that socio-economic differences in a broad range of health problems are already present early in life, and persist during childhood. Children from families with low socio-economic backgrounds experience more asthma symptoms (odds ratio (OR) 1.27; 95% Confidence Interval (CI) 1.08-1.49), poorer general health (OR 1.36; 95% CI 1.16-1.60), more frequent respiratory infections (OR 1.57; 95% CI 1.35-1.83), more overweight (OR 1.42; 95% CI 1.16-1.73), and more obesity (OR 2.82; 95% CI 1.80-4.41). The most important contributors to the observed childhood socio-economic health disparities are socio-economic differences in maternal age at birth, breastfeeding, and day-care centre attendance.

**Conclusions:**

Socio-economic health disparities already occur very early in life. Socio-economic disadvantage takes its toll on child health before birth, and continues to do so during childhood. Therefore, action to reduce health disparities needs to start very early in life, and should also address socio-economic differences in maternal age at birth, breastfeeding habits, and day-care centre attendance.

## Background

Those who are socio-economically better off are generally in better health [1]. This association between socio-economic status (SES) and health is very strong, and is consistent throughout life [2]. Even early in life, low family SES is related to low birthweight, poor cognitive test scores, and behavioural problems and socialization [3]. However, little is known about when socio-economic health differences set in during childhood and how these differences develop over time. Most studies on childhood SES and health use cross-sectional data. Only a few studies consider trajectories of changes in health over time [4].

The family environment is the first and most intimate level that influences the early development and health of children [5]. The family is important for nurturing, stimulating, and supporting children during the early years of life. Family-level characteristics (e.g. parental lifestyle, family resources, and parenting style) influence a family's nurturing and supporting competences [5]. Childhood socio-economic health disparities might be rooted in differences between socio-economic groups concerning a family's competence to create a healthy and stimulating environment for their offspring. Parents with lower SES more often behave in ways that are unhealthy for their children (e.g. smoking during pregnancy and continuing to do so afterwards, not breastfeeding, etc.) [6-8]. Furthermore, family skills for raising a child in a nurturing, stimulating, and supportive manner are related to the age of the mother. An increasing maternal age is positively related to the educational, behavioural, and mental health development of children [9]. The birthweight of the child is also related to the parents' SES, and is a risk factor for health in childhood [10]. Nowadays, women from a higher socio-economic background have their children more often at an older age than women from a lower socio-economic background [11].

Family size also seems to be relevant when it comes to the development of childhood health. Having siblings was found to protect children against hay fever, and, in several studies, against asthma and eczema as well [12]. It has been suggested that having siblings protects against atopy because of exposure to infections early in life, although this is still a subject of debate [12,13].

**Figure 1:**
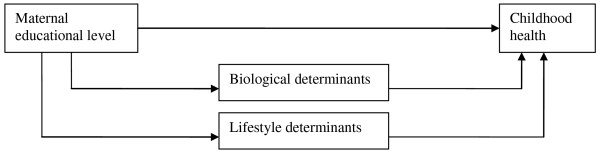
**Conceptual framework for the relationship between maternal educational level and children's health**.

This paper describes the development of socio-economic health differences early in life in the Netherlands. We studied the impact of maternal educational level (as an indicator of the socio-economic background of the child) on the development of several health indicators among a birth cohort until the children are eight years old. The health outcomes studied are common childhood health problems: eczema, asthma symptoms, general health, frequent respiratory infections, overweight, and obesity. We explored when socio-economic differences in health occur during childhood, and whether these health disparities change as children grow older. Furthermore, we investigated explanations for socio-economic disparities in children's health during the first eight years of their lives (Figure 1).

## Methods

### Study design and study population

Data were obtained from the Dutch longitudinal Prevention and Incidence of Asthma and Mite Allergy (PIAMA) study, which gathered health information on 3,963 children born in 1996 or 1997. The study collected health data during the first eight years of their lives, and is described in more detail elsewhere [14]. The study protocol was approved by the medical ethics committees of the participating institutes and all parents gave written informed consent. We have received approval from the PIAMA research group to use this data.

The parents filled in questionnaires during pregnancy, when the child was three months old, and subsequently, every year until the child was eight years old. Questionnaires were sent to the parents once a year in the month the child celebrated his or her birthday.

### Measures

#### Health outcomes

A child was classified as having eczema if an itchy rash had been present at one or more of the specified locations (the folds of the elbows or behind the knees, around the ears or eyes, or at the front of the ankles) during the previous 12 months. The question about eczema was asked every year at ages one to eight.

Children were categorized as having asthma symptoms if they had one or more symptoms: a wheeze, shortness of breath (dyspnoea) or if a medical doctor had prescribed inhaled steroids for respiratory or lung problems in the preceding 12 months. Asthma symptoms were reported annually starting at three years of age.

Frequent respiratory infections were measured annually from ages one to eight. Frequent respiratory infections were defined as having suffered from 3 or more of the following infections during the previous 12 months: bronchitis, pneumonia, middle ear infection, sinusitis, throat infection, or flu or a serious cold.

A Dutch version of the 'RAND General Health Rating Index' for children was used to assess general health [15]. The 'RAND General Health Rating Index' for children contains seven questions on general health and susceptibility to illness, and was asked at ages four, six, and eight. Answers were combined into one score between 7 and 32. The lowest quartile characterizes poor general health.

Overweight and obesity (severe overweight) were defined using weight and height information collected from the questionnaires at ages three to eight. Parents were asked to report their child's body weight (in kg) and height (in cm) from the last time the child was weighed and measured by a medical doctor or nurse if this had been done within the previous three months. If these measures were not available, the parents were asked to weigh and measure their child without shoes and heavy clothes. Age- and sex-specific cut-off points defined by Cole et al. [16] were used to classify the children as being overweight or obese. The health outcome overweight also includes obesity. The term 'weight problems' is used in here to describe both overweight and obesity.

#### Socio-economic status measure

Maternal educational level was used to indicate the family's socio-economic status (SES). Education is considered to be a good indicator of adult SES in the Netherlands and elsewhere [17,18]. It is an indicator of someone's knowledge, which is an important component of SES, alongside status and wealth. Also, education predicts other indicators of SES (income and occupation). In the one-year questionnaire, the question about educational level was asked in terms of the highest attained educational level, and divided into three categories: primary school or lower vocational or lower secondary education (low), intermediate vocational education or intermediate/higher secondary education (intermediate), and higher vocational education and university (high). Educational level was not reported by 156 mothers (4%).

#### Biological and lifestyle determinants

This study examined how biological and lifestyle factors may affect the development of health inequalities early in life. Determinants present at birth and in the child's first year were chosen to examine their influence on the development of health inequalities from birth onward. The biological and lifestyle determinants used in the explanatory analyses were selected on theoretical grounds. The research group included those determinants they considered most plausible and explanatory for SES differences in childhood health. The biological determinants were the presence of older siblings (yes/no), maternal age at birth (continuous), and the child's birthweight (continuous), and were reported in the questionnaire administered at three months. Lifestyle variables were reported in the child's first year: maternal smoking (during at least the first four weeks of the pregnancy), smoking by anyone in the home (more than once a week), breastfeeding, including any form of breastfeeding, also partial (never, 1-16 weeks, or > 16 weeks), and day-care centre attendance during the first year. Day-care centre attendance was dichotomized into 'none or fewer than 4 hours per week' and '4 hours per week or more'.

### Analyses

We used generalized estimating equations (GEE) to analyse the development of socio-economic health disparities during the first eight years of the children's lives. GEE models take into account the correlation between repeated measurements in the same individual. An autoregressive correlation was chosen to fit the data. All analyses used the most highly educated group as reference category, and were adjusted for the sex of the child.

First, we estimated the association between maternal educational level and the health outcomes for the whole period by fitting a GEE model with the child's age, sex and maternal educational level. Second, to find out whether educational differences in children's health varied during the period studied, we included the interaction between the child's age and maternal educational level in the model. Third, we studied which variables longitudinally predicted childhood health by fitting GEE models containing the child's sex, age, and one potential determinant. Variables were considered predictors of the health outcome if they were significantly associated with the health outcome (p < 0.05). Next, for those determinants significantly related to one or more health conditions, we studied the distribution of the predictor according to the educational level of the mother. Finally, if the predictors are also significantly related to the educational level of the mother (p < 0.05), we added the predictor to the basic GEE model (including the child's sex, age, and maternal educational level) in an attempt to explain the association between maternal educational level and childhood health. Time-dependent relationships between determinants and health outcomes were included in the model when significant, and we calculated the contribution of all significant determinants together to the educational differences in children's health. Determinants were excluded from the total model if we found no reduction in the odds ratios (ORs) and/or in the 95% confidence intervals (CIs) of the ORs of the different educational groups.

#### Missing data and multiple imputations

At eight years of age, 8% (310) of the 3,963 children included at baseline were lost to follow-up. In addition, not every parent always filled in all of the questions every year, which resulted in missing data. Particularly affected were the health outcomes, with missing data ranging from 15% for frequent respiratory infections to 35% for overweight and obesity. If data are not 'missing completely at random' (MCAR), complete case analysis may lead to biased results [19,20]. To overcome this problem, missing data were imputed multiple times for every year using the Multivariate Imputation by Chained Equations (MICE) procedure in the statistical program R (version 2.9.1), which resulted in five imputed datasets. Multiple imputation is a statistical technique that uses all observed data to fill in plausible values for the missing values [20]. In our case, this meant that the relationships between all variables used in the analyses were used to fill in the missing values in our dataset. SAS software (Statistical Analysis System, version 9.2*) *was used to perform Proc Genmod analyses and PROC MIANALYSE to pool the outcomes from the five datasets. All analyses described above were performed using both the original dataset and the imputed datasets. In this article, we report results from the analyses of the imputed datasets. The characteristics of the study population of the imputed dataset did not differ from the original dataset (Table 1). For all six outcome variables, imputation slightly increased the prevalence, indicating that children with no health problems are somewhat overrepresented in the complete cases. Also, the relationship between frequent respiratory infections among children and intermediate maternal educational level was statistically significantly in the imputed datasets but not in the original dataset. In addition, analyses of the imputed datasets led to conclusions that were similar to analyses of the original dataset.

**Table 1:** Characteristics of the study population of the original dataset and the imputed datasets.

	Baseline population Original data		Baseline population Imputed data
	**%**	**N**	**% (n = 3,963)**

Maternal educational level		3,807	
Low	23.5		23.8
Intermediate	41.6		41.7
High	35.0		34.5
			
Sex		3,963	
Female	48.1		48.1
Male	51.9		51.9
			
Older sibling(s)		3,937	
Yes	50.7		50.6
No	49.4		49.4
			
Maternal smoking during pregnancy^a^		3,904	
Yes	17.8		17.9
No	82.2		82.1
			
Breastfeeding duration		3,896	
No breastfeeding	17.9		18.0
1-16 weeks	49.6		49.7
>16 weeks	32.5		32.3
			
Smoking in the home		3,935	
Yes^b^	28.7		28.7
No	71.3		71.3
			
Day-care centre attendance		3,796	
>= 4 hours per week	24.4		24.2
<4 hours per week	75.6		75.9
			
Eczema at age 8	16.0	3,233	16.4
			
Asthma symptoms at age 8	13.0	3,308	13.8
			
Poor general health at age 8	24.6	3,229	26.0
			
Freq. respiratory infections at age 8	3.9	3,247	4.3
			
Overweight at age 8^c^	11.6	2,607	12.0
			
Obesity at age 8	2.0	2,607	2.3

	**Mean/SD^d^**		**Mean/SD^d^**

Maternal age (years)	30.3/3.9	3,920	30.3/3.9
Birthweight child (grams)	3,507/546	3,914	3,507/546

^a ^smoking by the mother during at least the first four weeks of pregnancy

^b ^smoking by anyone in the home more than once a week

^c ^overweight includes obesity

^d ^standard deviation

## Results

The prevalence of asthma symptoms decreases from 23% at the age of three to 13% at the age of eight. The prevalence of frequent respiratory infections decreases from 14% to 4% between the ages of three and eight. As the children grow older, more children become overweight or obese. At three years of age, 8% of the children are overweight and fewer than 1% are obese. At eight years of age, 12% are overweight and 2% are obese. The prevalence of poor general health and eczema fluctuated (Table 2).

**Table 2:** Prevalence (%) of health outcomes until age 8 and prevalence (%) of health outcomes at age 8 according to maternal educational level.

	Age								Maternal educational level^a^		
	**1**	**2**	**3**	**4**	**5**	**6**	**7**	**8**	**Low**	**Intermediate**	**High**

Eczema	15.3	16.8	17.6	18.2	15.6	15.9	14.9	16.4	17.0	16.5	15.9
Asthma symptoms	-	-	23.6	19.6	18.3	15.4	13.1	13.8	15.9	14.0	12.2
Poor health index	-	-	-	27.0	-	23.5	-	26.0	29.3	26.1	23.5
Freq. resp. infections^b^	14.6	15.0	12.1	10.6	10.7	8.4	4.8	4.3	5.3	4.2	3.8
Overweight ^c ^	-	-	7.4	9.5	10.1	10.6	11.8	12.0	16.0	11.6	9.6
Obesity	-	-	0.9	1.8	2.1	2.6	2.4	2.3	4.1	2.1	1.2

^a ^prevalence of health outcomes at age 8 according to educational level of the mother

^b ^frequent respiratory infections

^c ^overweight includes obesity

### Maternal education level and health outcomes

In general, children of mothers with lower educational levels are in poorer health than children of the most highly educated mothers (Tables 2 and 3). Educational differences in obesity were most prominent: children of mothers with the lowest educational level are obese 2.8 times more often than children of the most highly educated mothers. Already during childhood, an educational health gradient is present for asthma symptoms, poor general health, frequent respiratory infections, overweight, and obesity - that is, the lower the maternal educational level, the higher the health risk for the child. There are no educational differences in the prevalence of eczema (Table 3).

**Table 3:** Associations between maternal educational level and health outcomes at ages 0 to 8

	Health outcomes					
	**Eczema**	**Asthma** **symptoms**	**Poor general** **health**	**Frequent** **respiratory** **infections**	**Overweight^c^**	**Obesity**

**Maternal educational level**	**OR^a ^(CI^b^)**	**OR^a ^(CI^b^)**	**OR^a ^(CI^b^)**	**OR^a ^(CI^b^)**	**OR^a ^(CI^b^)**	**OR^a ^(CI^b^)**

Low	1.00 (0.86-1.17)	1.27 (1.08-1.49)	1.36 (1.16-1.60)	1.57 (1.35-1.83)	1.42 (1.16-1.73)	2.82 (1.80-4.41)
Intermediate	1.00 (0.88-1.14)	1.16 (1.00-1.34)	1.11 (0.96-1.27)	1.17 (1.02-1.34)	1.16 (0.96-1.40)	1.71 (1.11-2.63)
High	1.00	1.00	1.00	1.00	1.00	1.00

^a ^odds ratio of GEE analyses, adjusted for sex of the child

^b ^95% confidence intervals of the odds ratio

^c ^overweight includes obesity

### Trends according to age in the association between maternal educational level and health outcomes

**Figure 2:**
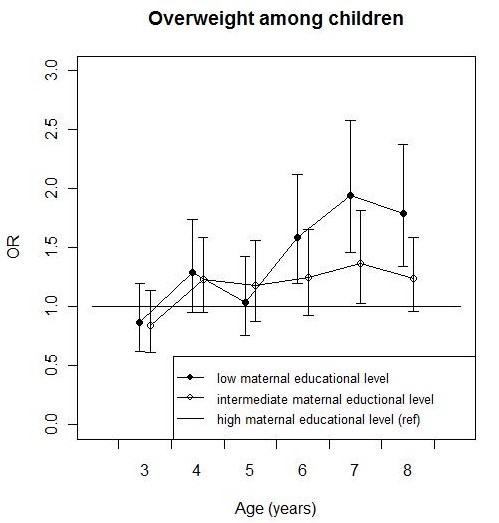
**Development of educational differences in overweight among children at ages 3 to 8, in odds ratios (ORs)^a^**. The development of children's overweight according to the educational level of the mother. Maternal educational level is divided into low, intermediate and high education. High maternal educational level is the reference category. ORs are presented with 95% confidence intervals. ^a ^Overweight includes obesity.

Except for overweight, the educational differences in health are not related to the age of the child and do not change over time. In the case of overweight, educational differences emerge at age six (Figure 2).

### Associations between the biological and lifestyle factors and health outcomes

Table 4 shows the results of GEE analyses that indicate which factors are associated with health during childhood. Having an older mother and a higher birthweight are protective factors against asthma symptoms, poor general health, and frequent respiratory infections in childhood. However, a higher birthweight increases the odds for overweight and obesity in childhood. Having siblings is positively related to frequent respiratory infections.

**Table 4:** Associations between biological and lifestyle factors and health outcomes at ages 0 to 8, in the total population.

	Asthma symptoms		Poor general health		Frequent respiratory infections		Overweight^r^		Obesity	
***Biological factors***	**OR^a^**	**(CI^b^)**	**OR^a^**	**(CI^b^)**	**OR^a^**	**(CI^b^)**	**OR^a^**	**(CI^b^)**	**OR^a^**	**(CI^b^)**

Siblings										
No	1.00		1.00		1.00		1.00		1.00	
Yes	1.14 (1.01-1.29)		0.90 (0.80-1.01)		1.15 (1.02-1.30)		1.08 (0.93-1.25)		1.00 (0.73-1.36)	
										
Maternal age at birth	0.98 (0.96-0.99)		0.95 (0.94-0.97)		0.98 (0.96-0.99)		0.99 (0.97-1.02)		0.99 (0.94-1.04)	
										
Birthweight	0.77 (0.68-0.87)		0.78 (0.70-0.88)		0.87 (0.77-0.97)		1.95 (1.69-2.25)		1.91 (1.29-2.82)	
										

***Lifestyle factors***										

Smoking during pregnancy										
No	1.00		1.00		1.00		1.00		1.00	
Yes ^c^	1.32 (1.11-1.57)		1.29 (1.11-1.50)		1.31 (1.13-1.52)		1.25 (1.02-1.53)		1.95 (1.36-2.80)	
										
Smoking in the home										
No	1.00		1.00		1.00		1.00		1.00	
Yes^d^	1.14 (0.99-1.32)		1.12 (0.98-1.29)		1.12 (0.98-1.28)		1.23 (1.03-1.48)		1.73 (1.23-2.42)	
										
Breastfeeding										
> 16 weeks	1.00		1.00		1.00		1.00		1.00	
1-16 weeks	1.37 (1.19-1.58)		1.21 (1.06-1.39)		1.28 (1.11-1.47)		1.08 (0.91-1.29)		1.44 (0.95-2.18)	
Never	1.52 (1.26-1.83)		1.32 (1.11-1.57)		1.60 (1.34-1.91)		1.25 (0.98-1.59)		1.81 (1.07-3.04)	
										
Day-care centre attendance										
Yes	1.00		1.00		1.00		1.00		1.00	
No ^e^	1.11 (0.96-1.29)		1.54 (1.31-1.81)		1.08 (0.93-1.24)		1.43 (1.16-1.78)		2.28 (1.45-3.58)	

^a ^odds ratio of GEE analyses, adjusted for sex

^b ^95% confidence intervals of the odds ratio

^c ^smoking by the mother during at least the first four weeks of pregnancy

^d ^smoking by anyone in the home more than once a week

^e ^no means < 4 hours per week

^f ^overweight includes obesity

Smoking during pregnancy increases the risk for all health problems mentioned in Table 4. When parents report smoking in the home at least once a week, their children are more likely to have weight problems (overweight and obesity). Breastfeeding for fewer than 16 weeks or not at all increases the risk of asthma symptoms, poor general health, frequent respiratory infections, and obesity in childhood. Furthermore, not attending day-care facilities in the first year of life increases the risk for poor general health, overweight, and obesity in childhood.

### Distribution of the biological and lifestyle factors according to educational level of the mother

Table 5 shows the distribution of the biological and lifestyle factors according to maternal educational level. Except for having siblings, all factors show an educational gradient (Table 5). Smoking during pregnancy, smoking in the home, not breastfeeding your child, and not attending day-care facilities are inversely related to maternal educational level. More highly educated mothers smoke less often during pregnancy and in the home, and they breastfeed their children more often and make more use of day-care facilities. Maternal age at birth and birthweight are positively related to the educational level of the mother, although the gradient is less clear here. Having siblings is more prevalent only in the lowest educational group when compared to the highly educated mothers. A sibling effect on health is therefore only expected for children of the mothers with the lowest educational level.

**Table 5:** Prevalence and means of biological and lifestyle factors of poorer health in childhood according to maternal educational level.

	Maternal educational level					
	**Low**		**Intermediate**		**High**	

***Lifestyle factors***	**%**	**(CI)^a^**	**%**	**(CI)^a^**	**%**	**(CI)^a^**

Smoking during pregnancy						
Yes ^b^	30.2	(29.1-31.2)	17.3	(16.6-17.9)	10.6	(10.1-11.2)
						
Smoking in the home						
Yes ^c^	43.5	(42.3-44.6)	30.6	(29.8-31.4)	16.2	(15.6-16.9)
						
Breastfeeding						
1-16 weeks	49.8	(48.6-50.9)	52.4	(51.5-53.3)	46.4	(45.4-47.3)
Never	30.6	(29.5-31.6)	18.4	(17.7-19.0)	8.5	(8.0-9.0)
						
Day-care centre attendance						
No^d^	88.9	(88.2-89.7)	79.7	(79.0-80.4)	62.5	(61.6-63.4)

***Biological factors***						

Siblings						
Yes	54.4	(53.3-55.5)	49.6	(48.7-50.4)	49.4	(48.5-50.3)

	**Mean**		**Mean**		**Mean**	

Maternal age at birth	29.55	(29.46-29.64)	29.78	(29.72-29.85)	31.59	(31.53-31.66)
						
Birth weight child (grams)	3,456	(3,443-3,469)	3,507	(3,498-3,516)	3,543	(3,532-3,553)

^a ^95% confidence intervals

^b ^smoking by the mother during at least the first four weeks of pregnancy

^c ^smoking by anyone in the home more than once a week

^d ^no means < 4 hours per week

### Explanations for educational differences in children's health

Table 6 shows the contribution of the biological and lifestyle factors to the relationship between educational level of the mother and childhood health. All factors together reduce the increased OR for asthma symptoms among children of mothers with low and intermediate educational levels to nearly 1 (OR 1.04), suggesting that the factors together largely contribute to the educational differences in childhood asthma. Breastfeeding contributes most to the educational differences in asthma symptoms. Mothers with lower educational levels are less likely to breastfeed their children, which increases the risk of asthma symptoms among the children. Also, smoking during pregnancy contributes substantially to the relationship between maternal educational level and asthma symptoms. Mothers with lower educational levels smoke more often during at least the first four weeks of their pregnancy, which increases their children's risk of asthma symptoms. Also, the age of the mother at birth contributes to the educational differences in asthma symptoms. Mothers with lower educational levels have their children at a younger age, and younger mothers report asthma symptoms more often.

**Table 6:** Associations between maternal educational level and health outcomes (ages 0 to 8), adjusted for biological and lifestyle factors.

	Asthma symptoms				Poor general health				Frequent respiratory infections			
**Educational level^c^**	**Intermediate**		**Low**		**Intermediate**		**Low**		**Intermediate**		**Low**	

	**OR^a^**	**CI^b^**	**OR^a^**	**CI^b^**	**OR^a^**	**CI^b^**	**OR^a^**	**CI^b^**	**OR^a^**	**CI^b^**	**OR^a^**	**CI^b^**

Model A	1.16	(1.00-1.34)	1.27	(1.08-1.49)	1.11	(0.96-1.27)	1.36	(1.16-1.60)	1.17	(1.02-1.34)	1.57	(1.35-1.83)
Model A + siblings	1.16	(1.00-1.34)	1.26	(1.07-1.48)	-		-		1.17	(1.02-1.34)	1.56	(1.34-1.82)
Model A + maternal age at birth	1.12	(0.97-1.30)	1.22	(1.03-1.44)	1.02	(0.86-1.17)	1.24	(1.05-1.46)	1.14	(0.99-1.30)	1.51	(1.29-1.78)
Model A + birthweight	1.15	(0.99-1.33)	1.24	(1.05-1.46)	1.10	(0.96-1.26)	1.34	(1.14-1.57)	1.17	(1.02-1.34)	1.55	(1.33-1.81)
Model A + smoking during pregnancy^d^	1.14	(0.99-1.32)	1.20	(1.02-1.42)	1.09	(0.95-1.26)	1.31	(1.11-1.54)	1.16	(1.01-1.33)	1.51	(1.29-1.76)
Model A + smoking in the home^e^	-		-		-		-		-		-	
Model A + breastfeeding	1.10	(0.95-1.27)	1.16	(0.98-1.37)	1.08	(0.93-1.24)	1.30	(1.09-1.54)	1.12	(0.98-1.28)	1.44	(1.22-1.69)
Model A + day-care centre attendance^f^	-		-		1.04	(0.91-1.19)	1.24	(1.05-1.45)	-		-	

Total ^g,h,1^	1.04	(0.90-1.21)	1.04	(0.88-1.24)	0.94	(0.82-1.08)	1.06	(0.90-1.26)	1.06	(0.93-1.22)	1.32	(1.11-1.57)

	**Overweight^l^**				**Obesity**							
				
**Educational level^c^**	**Intermediate**		**Low**		**Intermediate**		**Low**					
				
	**OR^a^**	**CI^b^**	**OR^a^**	**CI^b^**	**OR^a^**	**CI^b^**	**OR^b^**	**CI^b^**				
				
Model A	1.16	(0.96-1.40)	1.42	(1.16-1.73)	1.71	(1.11-2.63)	2.82	(1.80-4.41)				
Model A + siblings	-		-		-		-					
Model A + maternal age at birth	-		-		-		-					
Model A + birthweight	1.19	(0.99-1.44)	1.50	(1.23-1.82)	1.75	(1.14-2.69)	2.97	(1.91-4.61)				
Model A + smoking during pregnancy^d^	1.15	(0.95-1.39)	1.37	(1.12-1.67)	1.68	(1.10-2.56)	2.55	(1.61-4.02)				
Model A + smoking in the home^e^	1.14	(0.94-1.37)	1.36	(1.11-1.66)	1.61	(1.05-2.46)	2.53	(1.62-3.93)				
Model A + breastfeeding	-		-		1.63	(1.04-2.55)	2.61	(1.64-4.14)				
Model A + day-care centre attendance^f^	1.11	(0.91-1.34)	1.32	(1.07-1.61)	1.52	(1.00-2.32)	2.41	(1.54-3.80)				
				
Total^j,k^	1.09	(0.90-1.32)	1.26	(1.02-1.54)	1.42	(0.93-2.18)	2.04	(1.28-3.26)				

Note: Model A includes sex of child, age, and maternal educational level

^a ^odds ratio of health, adjusted for sex of the child

^b ^95% confidence interval of the odds ratio

^c ^highest educational level is reference

^d ^smoking by the mother during at least the first four weeks of pregnancy

^e ^smoking by anyone in the home more than once a week

^f ^< 4 hours per week

^g ^total model for asthma includes siblings, maternal age at birth, birth weight, smoking during pregnancy and breastfeeding

^h ^total model for poor general health includes maternal age at birth, birth weight, smoking during pregnancy, breastfeeding and day-care centre attendance

^i ^total model for frequent respiratory infections includes siblings, maternal age at birth, birth weight, smoking during pregnancy and breastfeeding

^j ^total model for overweight includes smoking during pregnancy, smoking in the home and day care centre attendance

^k ^total model for obesity includes smoking during pregnancy, smoking in the home, breastfeeding and day-care centre attendance

^l ^overweight includes obesity

When all relevant biological and lifestyle factors are included in the model, the elevated OR for poor general health for children of mothers with the lowest educational level compared to children of highly educated mothers reduces from OR 1.36 to OR 1.06 (Table 6). For children of mothers with an intermediate educational level, the elevated OR drops from OR 1.11 to OR 0.94. Maternal age at birth and day-care centre attendance are important contributors to the educational differences in poor general health (Table 6). Mothers with low educational levels have their children at a younger age than more highly educated mothers. At the same time, younger mothers more often assess their children's health as being poorer. Day-care centre attendance is negatively associated with poor general health, meaning that children who attend day-care facilities are less often in poor general health than children who do not attend such facilities, while children of the mothers with the low educational levels attend day-care facilities less often.

All relevant lifestyle and biological factors of frequent respiratory infections reduce the increased OR of frequent respiratory infections among children of mothers with an intermediate educational level by more than a half, and by slightly less for children of mothers with a low educational level (Table 6). Breastfeeding habits contribute most to the relationship between maternal educational level and frequent respiratory infections in childhood (Table 6). Including breastfeeding in the basic model (along with maternal educational level, age, and sex of the child) reduces the ORs for frequent respiratory infections for children of mothers with an intermediate educational level from OR 1.17 to OR 1.12, and for children of mothers with a low educational level from OR 1.57 to OR 1.44. Mothers with lower educational levels breastfeed their children less often, while breastfeeding for fewer than 16 weeks is associated with more frequent respiratory infections later in life. Also, maternal age at birth contributes to the educational differences in frequent respiratory infections among the children. Mothers with lower educational levels are younger when they give birth, and younger mothers report more frequent respiratory infections in their children.

The higher odds for overweight and obesity among children of mothers with low and intermediate educational levels decreases considerably when all relevant factors are included in the model (Table 6). For both overweight and obesity, day-care centre attendance is the most important contributor to educational differences in weight problems (Table 6). Day-care centre attendance is associated with fewer weight problems (overweight and obesity), whereas children of mothers with lower educational levels attend day-care facilities less often than children of the most highly educated mothers. Including day-care centre attendance in the basic model (Model A, Table 6), reduces the increased OR for overweight and obesity for children of mothers with a lower educational level by approximately 25%. Also, mothers with a lower educational background report smoking during pregnancy and smoking in the home more often than mothers in the highest educational group, while this increases the risk of weight problems among children later in life.

## Discussion

Socio-economic differences in health problems are already present very early in life, and persist during the first eight years of the lives of children in the Netherlands. This study confirms that early socio-economic health inequalities exist for a broad spectrum of health outcomes. Children from families with lower socio-economic backgrounds experience more asthma symptoms, poorer general health, more frequent respiratory infections, and more weight problems. Most of these early socio-economic health differences were stable during the period studied, except for educational differences in overweight, which emerged at age six. The most important determinants of the observed childhood socio-economic health disparities are socio-economic differences in maternal age at birth, breastfeeding, and day-care centre attendance.

Further elaboration of the results requires consideration of the strengths and limitations of this study. Our study's strengths are the large study population combined with a low attrition rate (around 8% during the whole study period). The repeated data collection over a period of eight years (starting at birth) allows for longitudinal analyses to study the development of children's health. The PIAMA study contains a broad range of different health outcomes that enables a more thorough study of the relationship between a family's socio-economic background and the health development of their children.

One of the study's limitations is that all of the information on health is self-reported by the parents. Self-reported information is prone to reporting bias, which could distort our findings, assuming this bias is related to SES. Another limitation is the selective availability of variables for studying explanations for educational differences in childhood health. We could only study the contribution of the available determinants to the educational health inequalities. These determinants were gathered with the intention of unravelling the aetiology of asthma and allergies [14]. Furthermore, 35% of the data on overweight and obesity is missing. This is considerable. We dealt with possible selection bias due to missing data by using multiple imputations of the missing data. This reduces the risk of selection bias. Also, it allowed us to make more efficient use of the data, because all available data was used in the analyses.

Maternal educational level is used in this study as an indicator of family socio-economic background. The educational level of the mother is a good proxy for social status in the Netherlands, and is supported by the literature [17,18]. It is a predictor for the knowledge people possess and for the income they are likely to gain. We repeated our analyses with paternal educational level and found similar results. Other indicators for family socio-economic background were not available in this study.

Our study reports a consistent pattern of poorer health outcomes among children from families with low socio-economic backgrounds. Although this relationship is found in other studies, several studies have discussed a reverse SES effect [4]. Chen et al. [4] suggest that the variation in study findings could be related to the age of the children being studied. In the case of asthma, Chen et al. describe how higher risks of asthma are reported among lower socio-economic groups until approximately nine years of age. After this, sometimes no relationship, or a reverse relationship, is found [4]. We found no socio-economic differences in childhood eczema, in contrast with several other studies, which report that eczema occurs less often among children from lower socio-economic backgrounds [21,22].

Our study shows that breastfeeding plays an important role in the development of childhood socio-economic differences for asthma symptoms and frequent respiratory infections. Mothers from lower socio-economic backgrounds are less likely to breastfeed their children [8], whereas breastfeeding protects children from several diseases [6,23]. Our findings support the importance of promoting breastfeeding, especially among mothers with low socio-economic positions.

In this study, day-care centre attendance during the first year is related to socio-economic differences in children's poor general health and weight problems, but not to respiratory infections or asthma. On the one hand, day-care centre attendance is considered a threat to children's health (in particular with regard to respiratory infections) because of the close contacts between children in day-care centres [24,25]. Reports on the effects of day-care centre facilities on asthma are inconclusive [26,27]. On the other hand, day-care attendance is considered a stimulus for children's IQ, and has had beneficial effects on behavioural development and school achievement in the USA [28]. Studies examining the relationship between day-care centre use and overweight in childhood have found mixed results [29-33]. The protective health effect of day-care attendance found in our study might reflect other characteristics of families using day-care facilities that protect against ill health. In our study, the parents of children who attend day-care during their first year are more often both employed, and less often overweight. To truly disentangle the underlying mechanisms, more research is needed on the influence of day-care centre facilities on health.

Maternal age at birth is an important factor for childhood socio-economic differences in asthma symptoms, poor general health, and frequent respiratory infections. Younger mothers report poorer general health, more asthma symptoms, and more respiratory infections than older mothers. The reason for this might be that younger mothers are more worried than older mothers [34]. On the other hand, children of younger mothers might truly be unhealthier than their peers who have older mothers, for example, because younger mothers lack sufficient knowledge to nurture, stimulate, and support their children and stimulate their health [9].

In our study, mothers from low socio-economic groups smoke more often during pregnancy, which is associated with asthma and weight problems in childhood. For asthma, similar results were found in other studies [35,36]. Smoking during pregnancy was defined as smoking during the first four weeks. After that time, some mothers stopped smoking, while others did not [37]. We consider this variable to be an indicator for whether the mother was smoking when she became pregnant. The analyses show that this indicator is related to detrimental health effects for the child. We suggest that women stop smoking even before they get pregnant, for instance, when they stop using birth control. In addition, children of low SES parents are more likely to be exposed to environmental tobacco smoking in the home environment [38,39]. This is associated with weight problems in childhood. Smoking campaigns should pay more attention to these socio-economic inequalities, because this is an important risk factor for childhood health inequalities.

## Conclusions

This study confirms that socio-economic health disparities already occur very early in life. Socio-economic disadvantage takes its toll before birth and continues to do so during childhood. Therefore, action to reduce health disparities needs to start very early on in life. These findings fit in with the trend of intervening in the preconception phase, for instance, with health promotion activities to reduce risky behaviour among prospective parents [40,41]. More attention is needed to find effective ways of promoting a healthier lifestyle among parents and prospective parents from lower socio-economic backgrounds so that health disparities throughout the life course can eventually be tackled.

## Competing interests

The authors declare that they have no competing interests.

## Authors' contributions

AR performed the analyses and the writing of the manuscript. MD conceived the idea for this article, supervised the analyses and preparation of the manuscript, and reviewed the drafts of the manuscript. AHW was involved in the analyses and interpretation of the findings and reviewed the drafts of the manuscript. MK, GHK and JAS participated in the realization of the PIAMA birth cohort study and reviewed the final draft of the manuscript. All authors read and approved the final manuscript.

## Pre-publication history

The pre-publication history for this paper can be accessed here:

http://www.biomedcentral.com/1471-2458/11/225/prepub
